# Spin-transfer torque generated in graphene based topological insulator heterostructures

**DOI:** 10.1038/s41598-018-22680-4

**Published:** 2018-03-12

**Authors:** Qingtian Zhang, K. S. Chan, Jingbo Li

**Affiliations:** 1 School of Materials and Energy, Guangdong University of Technology, Guangzhou, Guangdong, 510006 People’s Republic of China; 2 Department of Physics, City University of Hong Kong, Tat Chee Avenue, Kowloon, Hong Kong, People’s Republic of China; 3 City University of Hong Kong Shenzhen Research Institute, Shenzhen, 5183000 People’s Republic of China

## Abstract

We studied the spin-transfer torque (STT) in graphene based normal-metal/topological-insulator/ferromagnet heterostructures (N/TI/F), which is induced by the helical spin-polarized current in the quantum spin Hall insulator. We found that the STT is comparable in magnitude to the STT in ferromagnetic-normal- ferromagnetic graphene junction, while not requiring additional ferromagnetic layer with fixed magnetization, which makes it advantageous for the manipulation of magnetic devices in spintronics. More interestingly, the STT is very robust in our proposed nanostructure, as it is immune to changes in the geometry due to an asymmetrically notch or the presence of random nanopores in the quantum spin Hall insulator. Our theoretical prediction suggests that graphene based quantum spin Hall insulator could be used for very efficient magnetization manipulation for magnetic materials.

## Introduction

The topological insulator is a new quantum state of matter characterized by an insulating bulk gap and gapless edge states topologically protected, which has been intensively studied owing to the new physical properties and potential technological applications^1–11^. The two-dimensional topological insulator, known as the quantum spin Hall insulator, was first predicted by Kane and Mele^12^ in graphene. The electrons can be transported in the gapless edge states while the bulk is insulating, and spin-polarized dissipationless currents are transported in the transport channels confined at the two edges without backscattering. However, the quantum spin Hall insulator can not be realized in graphene owing to the exceedingly weak spin-orbit interaction^13–16^. Recently, theoretical and experimental work suggested that spin-orbit interaction can be enhanced by appropriate substrates or adatom deposition^17–25^ which leads to the transition into the topological non-trivial phase. It is therefore useful to investigate how the topological non-trivial phase can be used in spintronic applications.

Spin-transfer torque (STT) is an important spintronic phenomenon, in which a spin current injected into a ferromagnetic layer exerts a torque on the magnetic layer and may change its magnetization orientation^26^. When spin current flows into a ferromagnetic layer, the magnetization of the ferromagnetic layer exerts a torque on the electron spins and the spin current exerts an equal but opposite torque on the ferromagnetic layer, so the orientation of magnetization is modified^27^. This spintronic effect was predicted independently by Slonczewski^28^ and Berger^29^, and it has been confirmed in many experiments^30–36^. In spintronics, it is of great importance to reorient the magnetizations in magnetic memory and logic devices, and the STT effect offers us an efficient method to control magnetizations through current. The STT effect can be used to manipulate the magnetic device elements without external magnetic fields.

Graphene has been proven to be a promising candidate for future practical applications in spintronics owing to the long spin relaxation time and length^37–39^ and the nonlocal electrical spin transport at room temperature was first demonstrated in graphene^40^. Some work has been done to study the STT generation in graphene based F/N/F structure. Ding *et al*.^41^ investigated theoretically the current-induced STT in ferromagnetic-normal-ferromagnetic graphene nanoribbon junction, and Yokoyama *et al*.^42^ also considered the STT generation in ferromagnetic-normal-ferromagnetic bulk graphene junctions.

Motivated by the successful idea of realizing quantum spin Hall insulator in graphene and the recent measurements of the STT induced by a topological insulator^30^, we theoretically study the generation of the STT in graphene nanoribbon through a quantum spin Hall insulator. It is found that reliable STT can be generated by a graphene based N/TI/F heterostructures, and the STT can not be affected by asymmetrically notched the ribbon or nanopores.

## Methodology

**Figure 1:**
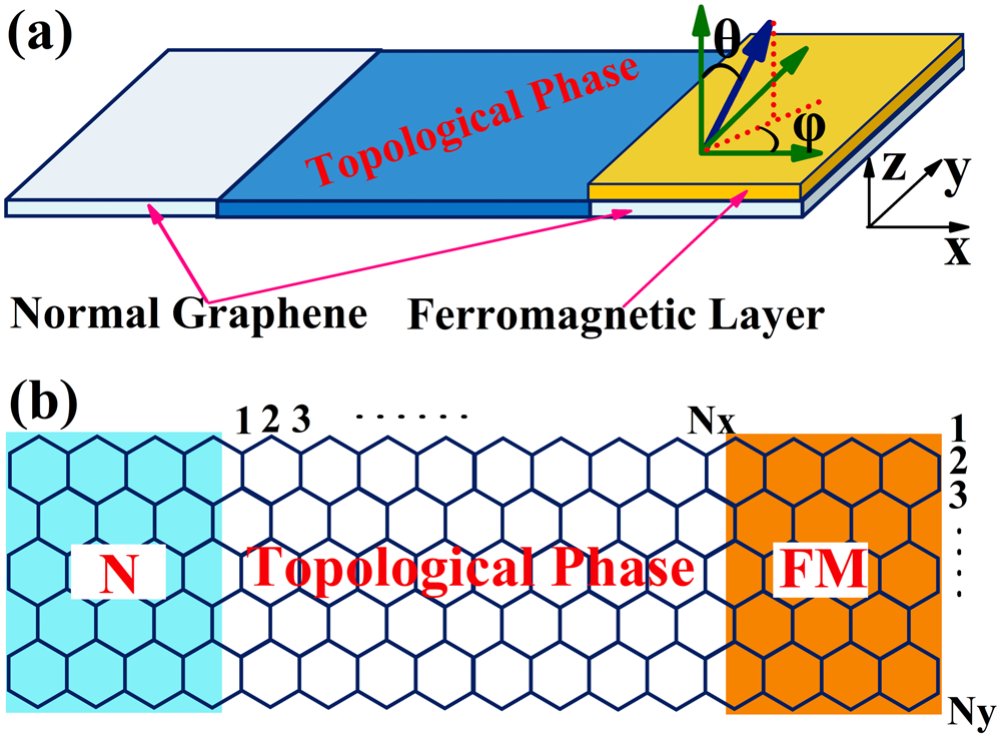
(**a**) Schematic illustration of the N/TI/F heterostructures: The graphene based topological insulator is connected to a normal graphene electrode and a ferromagnetic electrode. The magnetization direction (denoted by blue arrow) of the ferromagnetic electrode is assumed to be tunable. (**b**) Schematic view of the graphene nanoribbon device with a graphene based topological insulator connected with two semi-infinite leads. The size of the scattering region is determined by the ribbon length *N*_*x*_ and ribbon width *N*_*y*_.

The schematic view of the device under consideration is presented in Fig. 1, which consists of a graphene based quantum spin Hall insulator and two semi-infinite leads. The left lead is a normal graphene nanoribbon, and the right lead is a graphene nanoribbon with a ferromagnetic layer. The magnetization direction of the ferromagnetic layer is tunable and is described by the angle θ and φ. In Fig. 1(b), we show some information of the device considered in our calculation. In the scattering region, we consider a zigzag graphene nanoribbon in the topological phase obtained by including intrinsic spin-orbit interaction. The graphene based topological insulator is connected to the left normal graphene nanoribbon and the right ferromagnetic electrode. The total Hamiltonian for our N/TI/F structure is given by

1 $$H={H}_{N}+{H}_{TI}+{H}_{F}+{H}_{C}$$

where *H*_*N*_, *H*_*TI*_ and *H*_*F*_ are Hamiltonians for the normal graphene, graphene based topological insulator and ferromagnetic graphene respectively. In the tight-binding approximation, the Hamiltonians for the three different regions can be written as

2 $$\begin{array}{c}{H}_{N}=\sum _{i\alpha }\varepsilon {c}_{i\alpha }^{+}{c}_{i\alpha }-t\sum _{\langle ij\rangle \alpha }{c}_{i\alpha }^{+}{c}_{j\alpha }\\ {H}_{TI}=\sum _{i\alpha }\varepsilon {c}_{i\alpha }^{+}{c}_{i\alpha }-t\sum _{\langle ij\rangle \alpha }{c}_{i\alpha }^{+}{c}_{j\alpha }+i{\lambda }_{SO}\sum _{\langle \langle ij\rangle \rangle \alpha \beta }{v}_{ij}{c}_{i\alpha }^{+}{\sigma }_{\alpha \beta }^{z}{c}_{j\beta }\\ {H}_{F}=\sum _{i\alpha }[(\varepsilon +\alpha M\,\cos \,\theta ){c}_{i\alpha }^{+}{c}_{i\alpha }+M\,\sin \,\theta (\cos \,\phi -i\alpha \,\sin \,\phi ){c}_{i\alpha }^{+}{c}_{i\bar{\alpha }}]-t\sum _{\langle ij\rangle \alpha }{c}_{i\alpha }^{+}{c}_{j\alpha }\end{array}$$

where *ε* is the onsite energy, ${c}_{i\alpha }^{+}({c}_{i\alpha })$ denotes the creation (annihilation) operator of an electron with spin α at site i, t is the nearest neighbor hopping in the honeycomb lattice, $\langle ij\rangle (\langle \langle ij\rangle \rangle )$ denotes the summation over the nearest neighbor sites (the next nearest neighbor sites), *λ*_*SO*_ is the spin-orbit interaction, M is the magnetization of the ferromagnetic electrode, θ and φ describes the direction of the magnetization, *v*_*ij*_ = +1(−1) if the electron makes a counterclockwise (clockwise) turn, with respect to the positive z axis, in going from site j to site i. The coupling between the leads and the TI region is represented by the operator

$${H}_{C}=\sum _{\langle rs\rangle \alpha }-t({c}_{r\alpha }^{+}{c}_{s\alpha }+{c}_{s\alpha }^{+}{c}_{r\alpha }),$$

where t is the nearest-neighbor hopping energy, which is identical to other regions, and indices r and s denotes the sites in the TI and lead regions respectively. For example, r denotes sites in TI and s denotes sites in the leads.

For a small bias voltage V and zero temperature, the STT per unit of the bias voltage can be obtained using the nonequilibrium Green’s function method as^41,43^,

3 $$\frac{{\tau }^{Rx}}{V}=\frac{e}{4\pi }Tr\{[{G}^{r}({E}_{F}){{\rm{\Gamma }}}_{L}({E}_{F}){G}^{a}({E}_{F}){{\rm{\Gamma }}}_{R}({E}_{F})]({\sigma }_{x}\,\cos \,\theta -{\sigma }_{z}\,\sin \,\theta )\}$$

Here the ferromagnetic magnetization is in the x-z plane. This equation gives the in-plane torque which lies in the x-z plane and is obtained by considering the rate of change of the magnetization in the FM lead. When the FM magnetization is rotated by an angle φ about the z-axis, the in-plane torque becomes

4 $$\frac{{\tau }_{in}^{R}}{V}=\frac{e}{4\pi }Tr\{[{G}^{r}({E}_{F}){{\rm{\Gamma }}}_{L}({E}_{F}){G}^{a}({E}_{F}){{\rm{\Gamma }}}_{R}({E}_{F})]({\sigma }_{x}\,\cos \,\phi \,\cos \,\theta -{\sigma }_{y}\,\sin \,\phi \,\cos \,\theta -{\sigma }_{z}\,\sin \,\theta )\}$$

There is an out-of-plane torque which is perpendicular to the plane defined by the z-axis and the magnetization, which is given by the expression

5 $$\frac{{\tau }_{out}^{R}}{V}=\frac{e}{4\pi }Tr\{[{G}^{r}({E}_{F}){{\rm{\Gamma }}}_{L}({E}_{F}){G}^{a}({E}_{F}){{\rm{\Gamma }}}_{R}({E}_{F})]({\sigma }_{x}\,\sin \,\phi -{\sigma }_{y}\,\cos \,\phi )\}$$

These expressions describe the STT exerted on the magnetization of the ferromagnetic electrode by the electrons injected from N region and spin polarized when travelling through the topological insulator region. Using the same noequilibrium Green’s function (NEGF) formalism, the conductance can be obtained within the Landauer-Buttiker framework, and expressed as^44^

6 $$G({E}_{F})=\frac{{e}^{2}}{h}Tr[{{\rm{\Gamma }}}_{R}({E}_{F}){G}^{r}({E}_{F}){{\rm{\Gamma }}}_{L}({E}_{F}){G}^{a}({E}_{F})]$$

where ${{\rm{\Gamma }}}_{L(R)}=i[{{\rm{\Sigma }}}_{L(R)}^{r}-{{\rm{\Sigma }}}_{L(R)}^{a}]$ is written in terms of the L(R) lead self-energies ${{\rm{\Sigma }}}_{L(R)}^{r}$, and ${{G}}^{r(a)}({E}_{F})$ is the retarded (advanced) Green’s function. This expression is also valid for small bias and zero temperature as Eq. (3). All numerical calculations were performed using the Kwant tight-binding code^45^. Kwant is a Python package which can be used to calculate quantities, such as the Green’s function and the scattering matrix, and determine the transport properties of tight-binding models. The technical details about how to use Kwant can be found in ref.^45^. Here we give a brief description of the steps we used to find the Green’s function and the self-energies using Kwant. First of all, we constructed the tight-binding model of the scattering region using the Kwant package by specifying the atomic sites of the scattering region, the site energies of the sites and the hopping strengths between sties. This is carried out in the form of a small Python program. The spin degree of freedom of the atomic sites is represented by 2 × 2 matrices. For example, the spin-independent site energy and neighboring sites hopping are represented by the two 2 × 2 matrices $$[\begin{array}{cc}\varepsilon  & 0\\ 0 & \varepsilon \end{array}]$$ and $$[\begin{array}{cc}-t & 0\\ 0 & -t\end{array}]$$ respectively. The tight-binding model of infinitely long leads are constructed by providing the site positions along the transverse direction of the lead and the translation vector used to build the lead as well as the site energies and hopping between sites. After the tight-binding model of the structure is constructed using Kwant from the atomic sites, Kwant libraries are called in the program to calculate the Green’s function and the self-energies. The conductance and SST are obtained using the above expressions and the Green’s function and the self-energies obtained using Kwant.

## Results and Discussion

**Figure 2:**
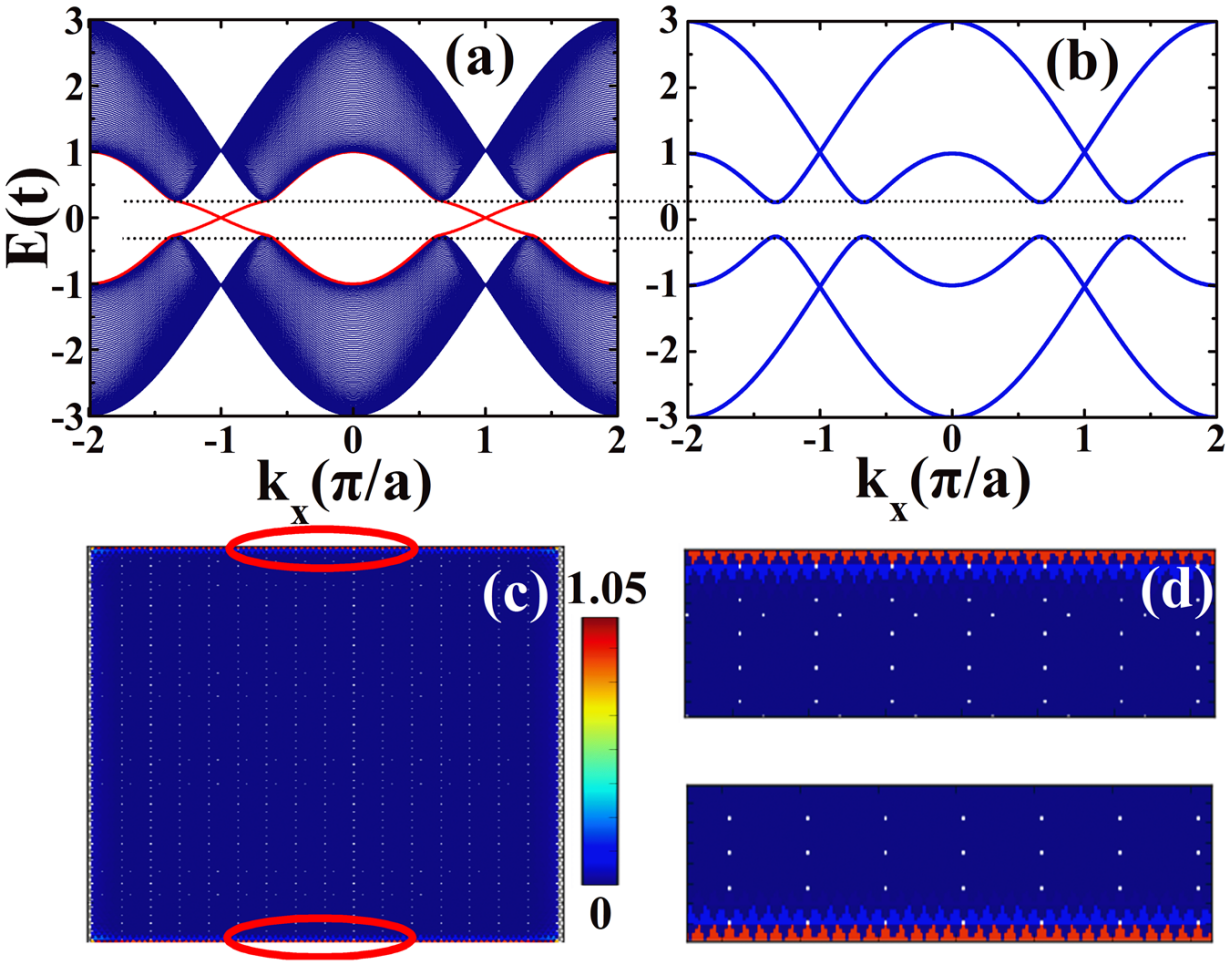
(**a**) Band structure of a zigzag graphene nanoribbon with spin-orbit interaction. (**b**) The corresponding bulk band structure along the line of *k*_*y*_ = 0. (**c**) The local density of states for the N/TI/F junctions when the Fermi energy is in the band gap. (**d**) The enlargement of the parts of the figure marked with red circles in (**c**). The parameters are: *E*_*F*_ = 0.1*t*, *λ*_*SO*_ = 0.05*t*, *M* = 0.01*t*, *N*_*x*_ = 160, *N*_*y*_ = 200, *θ* = 2*π*/3 and *φ* = 0.

It has been predicted by Kane and Mele^12^ that graphene exhibits a quantum spin Hall effect in the presence of intrinsic spin-orbit interaction. The spin-orbit interaction can be described by a second neighbor tight-binding model (see Eq. (2)), which will generate an energy gap in zigzag graphene nanoribbon. The edge states can be seen in the band structures for graphene in a stripe geometry in Fig. 2. In Fig. 2(a,b) we show the band structures for graphene nanoribbon and bulk graphene respectively. For the graphene nanoribbon considered, the ribbon width is set as *N*_*y*_ = 200, which is about 21.2 nm. It is noted in Fig. 2(b) that there are bulk bandgaps at the K and K’ points. However, we can also see that two bands (red lines in Fig. 2(a)) traverse the bulk gap, and connect the K and K’ points. These bands are topologically protected edge states, and they only localize at the two edge of the graphene nanoribbon. The spatial distribution of the local density of states is presented in Fig. 2(c,d), and Fig. 2(d) is the enlargement of the parts of Fig. 2(c). When the Fermi energy locates at the bulk energy gap, electrons can only transport through the edge of graphene nanoribbon. It is clear to see that the local density of states is high at the edge of the ribbon, but zero in the bulk.

**Figure 3:**
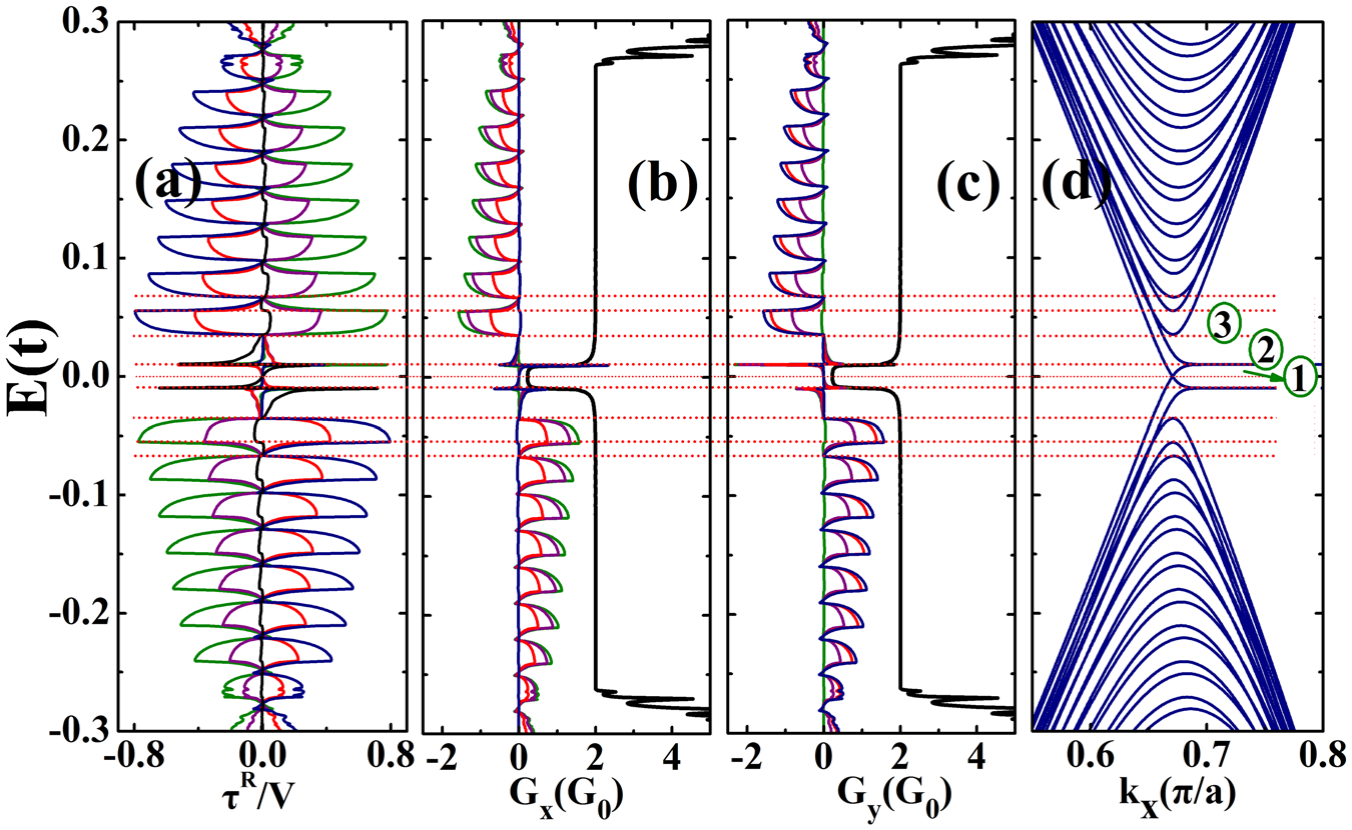
(**a**) The in-plane STT per unit of the bias voltage *V* in unit of *e*/4*π* for *φ* = 0(green), *φ* = *π*/6(purple), *φ* = *π*/3(red), *φ* = *π*/2(blue). The out-of-plane STT are the same for all the angle *φ* (black). (**b**) and (**c**) The total conductance (black) and the spin conductance plotted as a function of Fermi energy for *φ* = 0(green), *φ* = *π*/6(purple), *φ* = *π*/3(red), *φ* = *π*/2(blue). (**d**) The band structure of the right ferromagnetic graphene electrode. The other parameters are: *λ*_*SO*_ = 0.05*t*, *M* = 0.01*t*, *N*_*x*_ = 160, *N*_*y*_ = 200 and *θ* = 2*π*/3.

Figure 3(a) presents the current induced in-plane and out-of-plane STT per unit of the bias voltage, and it is plotted as a function of Fermi energy. It is found that the quantum spin Hall insulator can be used to generate in-plane STT on the ferromagnetic layer, and the magnitude of the STT oscillates with the Fermi energy. The dependence of STT on the Fermi energy can be understood from the spin conductance and the band structure of the right ferromagnetic graphene electrode (see Fig. 3(d)). The total conductance and spin conductances for x-polarized electrons and y-polarized electrons are presented in Fig. 3(b,c). In the figure, *G*_0_ = *e*^2^/*h*, which is the unit for conductance. The spin conductance is defined by ${G}_{m}=\frac{{e}^{2}}{h}Tr[{\sigma }_{m}{G}^{r}({E}_{F}){{\rm{\Gamma }}}_{L}({E}_{F}){G}^{a}({E}_{F}){{\rm{\Gamma }}}_{R}({E}_{F})]$ with *m* = *x*, *y*, *z* which is a very important quantity for the spin-dependent quantum transport. The STT can be expressed in terms of the spin conductances, for example, for the in-plane STT $\frac{{\tau }_{in}^{R}}{V}=\frac{\hslash }{2e}({G}_{x}\,\cos \,\phi \,\cos \,\theta -{G}_{y}\,\sin \,\phi \,\cos \,\theta -{G}_{z}\,\sin \,\theta )$. So, we also show *G*_*x*_ and *G*_*y*_ in Fig. 3 to show the relation between the quantities. *G*_*z*_ is very small and thus is not shown in the figure. Moreover, it can be noted in Fig. 3(a) that the STT is inversion symmetrical with respect to the axis of *E*_*F*_ = 0, which is caused by the electron-hole symmetry in graphene nanoribbons. The out-of-plane STT is negligibly small compared with the in-plane STT. About the dependence of the STT on the Fermi energy, firstly, it is noted that the STT in the energy range labeled by 1 in the figure is negligible, because the spin conductances are very small. In the energy range 2, the STT is zero. We can note that the spin conductances are close to zero in this range, which leads to a zero STT. In energy range 3, it is obvious that the x and y spin conductance are not negligible and the strong spin polarization causes a large STT. We want to point out that the in-plane STT obtained in this study is comparable in magnitude to the STT in a previous study, which was investigated in a ferromagnetic-normal-ferromagnetic graphene junction^41^. In Fig. 3, the angle φ dependence of STT and the spin conductances are also shown. It can be noted that when φ increases from 0 to π/2, *G*_*x*_ decreases from the highest value to 0, while *G*_*y*_ increases from 0 to the highest value. These changes cause the in-plane STT to change from a positive value to a negative value (reverse of direction). When the Fermi energy moves away from 0, the STT oscillates with the spin conductances. Zero STT is obtained when the Fermi energy coincides with a subband edge in the FM lead. The oscillation amplitude of the STT decreases with the Fermi energy, because the spin conductances decrease with increasing Fermi energy when there are more subbands in the FM lead into which the electrons can be scattered.

When the Fermi energy is outside the gap of TI (*E*_*F*_ > 0.25*t* or *E*_*F*_ > −0.25*t*), it can be noted in Fig. 3(a,b) and (c) that the spin conductances and STT further decrease, while the charge conductance increases. When the Fermi energy is outside the TI gap, the bulk subbands in the TI can carry current and thus the charge conductance increases. However, when there are more subbands, into which the electrons can be scattered, the spin conductances as well as the STT decrease. This trend agrees with the trend found for Fermi energies within the gap.

According to ref.^46^, the SOI strength is between 50 meV and 500 meV depending on the method of producing SOI. The present value of SOI strength is 140 meV (0.05t, with t = 2.8 eV) which is within this range. We have also considered other two values of strength within the range (280 meV and 56 meV). The results are similar to the present at most of the energies.

**Figure 4:**
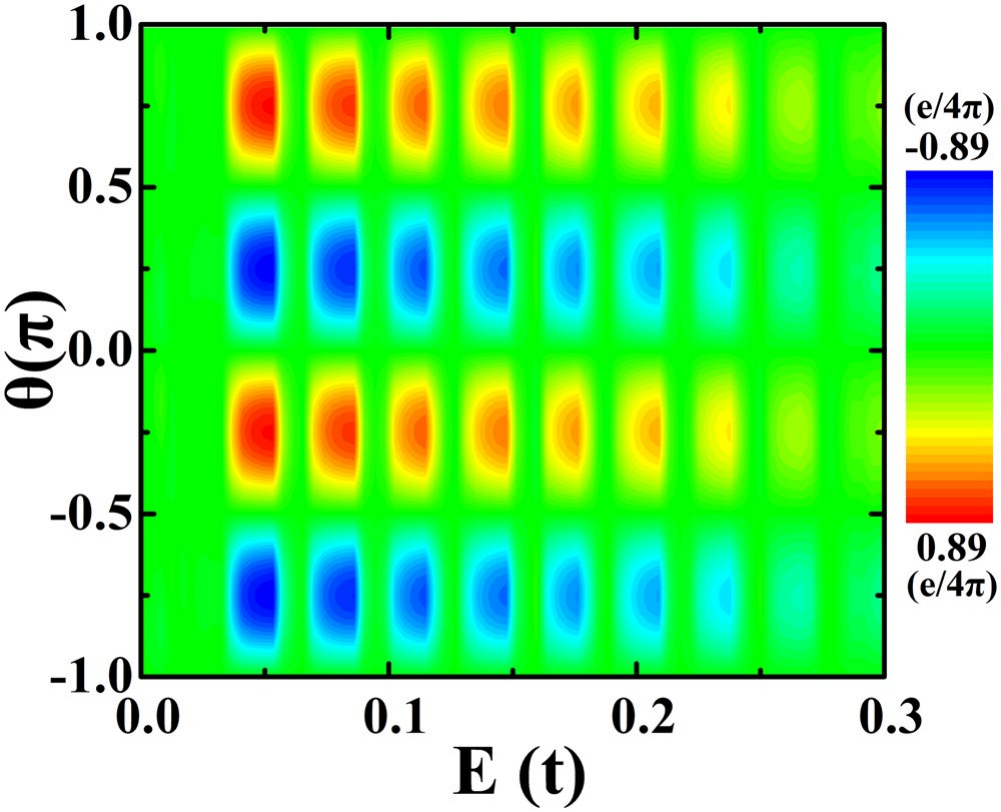
The contour plot of the in-plane STT per unit of the bias voltage *V* in unit of *e*/4*π*. The in-plane STT is plotted as a function of Fermi energy and the angle of the magnetization directions of the right ferromagnetic electrode. The other parameters are: *λ*_*SO*_ = 0.05*t*, *M* = 0.01*t*, *N*_*x*_ = 160, *N*_*y*_ = 200 and *φ* = 0.

In Fig. 4, the in-plane STT is plotted as a function of the Fermi energy and the direction angle θ of the magnetization for the ferromagnetic electrode. First, we can note that the STT around zero Fermi energy is zero, which is already explained in the discussion for Fig. 3. We can see that high STT is found at −π/4 and 3π/4, while low STT exists at −3π/4 and π/4. We can observe that the STT oscillates with the Fermi energy, and we can also see that the amplitude of STT decreases with an increase in Fermi energy. When the Fermi energy is larger than 0.25t, the Fermi energy is outside the gap of TI and the STT is very weak. This behaviour has been discussed in the discussion of Fig. 3 above.

**Figure 5:**
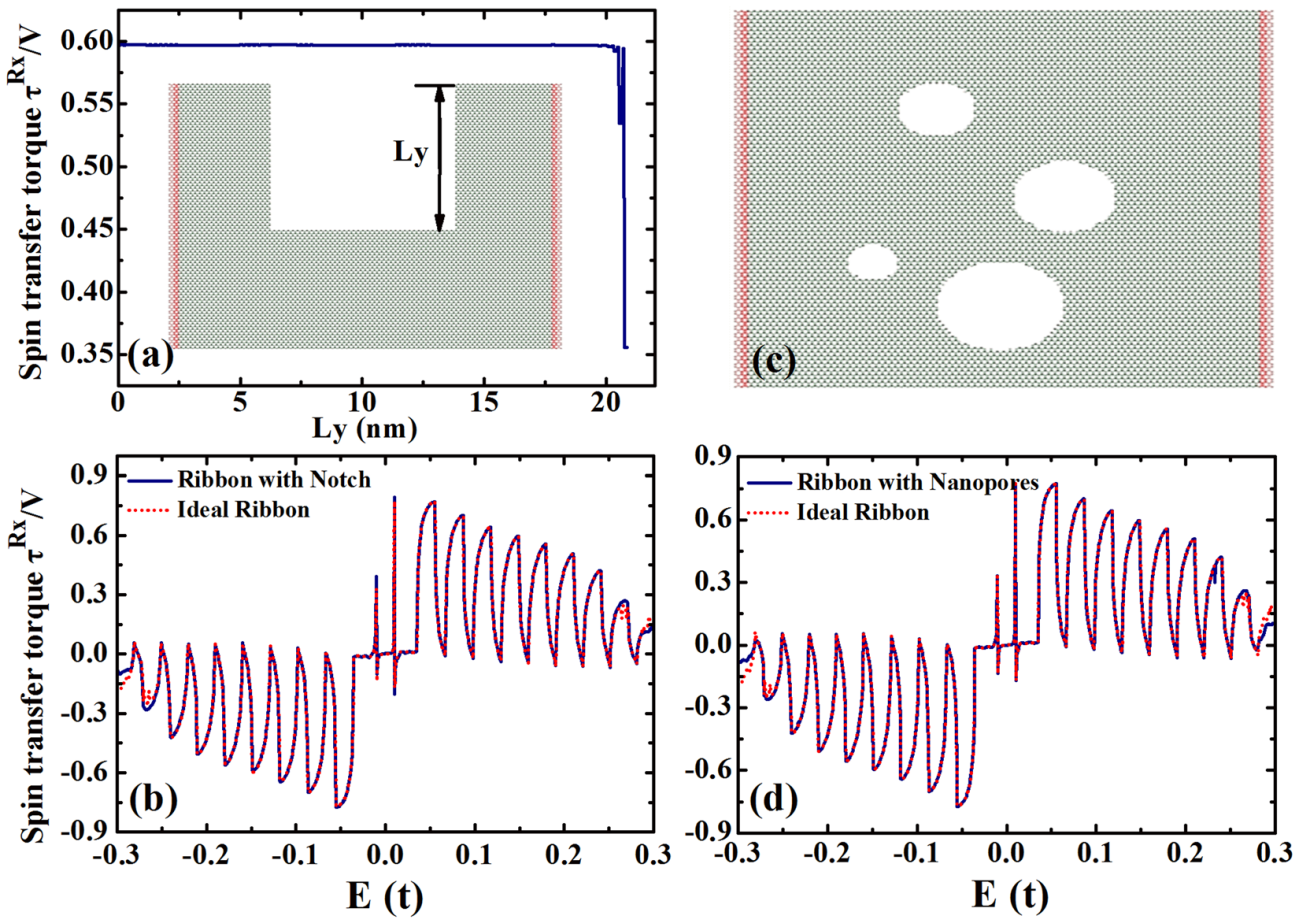
(**a**) The STT per unit of the bias voltage *V* in unit of *e*/4*π* is plotted as a function of the width of the square notch for graphene based topological insulator in the scattering region, and the inset is the schematic illustration of the device: Zigzag graphene nanoribbon with an asymmetric square notch. (**b**) The in-plane STT plotted as a function of energy for both ideal ribbon and notched ribbon. (**c**) Schematic illustration of the device: Zigzag graphene nanoribbon with nanopores. (**d**) The in-plane STT plotted as a function of energy for both ideal ribbon and naopored ribbon. The other parameters are: *λ*_*SO*_ = 0.05*t*, *M* = 0.01*t*, *N*_*x*_ = 160, *N*_*y*_ = 200, *θ* = 2*π*/3 and *φ* = 0.

Figure 5(a) shows the STT as a function of the width (*L*_*y*_) of the square notch for the graphene based topological insulator, and the inset is a schematic illustration of the devices in our numerical calculation. *L*_*y*_ is the width of the square notch, and we have an ideal ribbon when *L*_*y*_ = 0. In our calculation, the width of the ribbon is chosen to be *N*_*y*_ = 200 which is around 21.2 nm. Here, the width of the square notch changes from 0 to 20.8 nm. As shown in the figure, the STT does not change much with the width of the notch until it is very close to the ribbon width. To be more specific, we calculate the STT for a fixed notched square *L*_*y*_ = 10.2 *nm*, and it is plotted as a function of the Fermi energy (shown in Fig. 5(b)). We can see in Fig. 5(b) that the STT for a ribbon with a notch and an ideal ribbon are the same. That is because the topologically protected edge states can not be affected by the asymmetric square notch. When we have a notch in the scattering region, the electrons can still move from the left electrode to the right lead trough the helical edge channels. Figure 5(c) displays the schematic illustration of the device with random nanopores. We compare the STT of the device with nanopores with that of an ideal ribbon, and it is obvious that the nanopores do not affect the STT that generated by the graphene based topological insulator. Therefore, we can obtain reliable STT by a graphene based topological insulator, which is not affected by the geometry of the scattering region.

We have also considered structures with armchair edges and random edges, which consist of random combination of armchair and zigzag edges with random lengths. The random edges are created by cutting triangles of random sizes from a zigzag edged structure. The STT obtained in these structures are close to the zigzag edged structure for low E_F_ (<0.25t).

**Figure 6:**
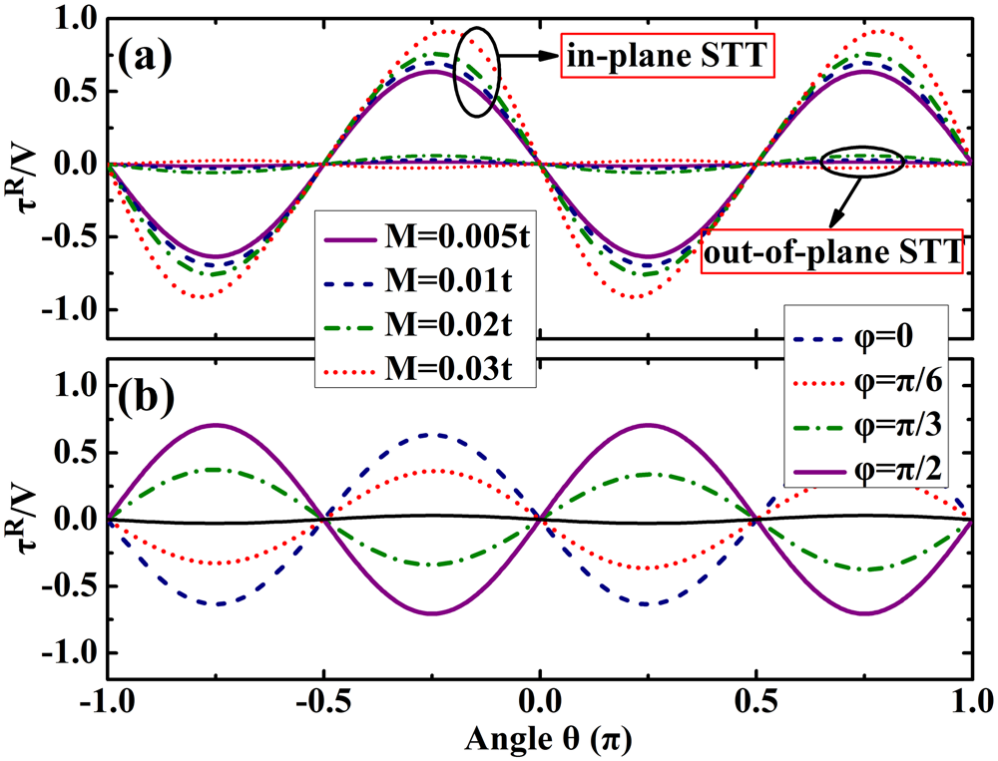
(**a**) The in-plane and out-of-plane STT plotted as a function of the direction angle θ of the magnetization for different magnetization intensity for *φ* = 0. (**b**) The in-plane STT is plotted as a function of the direction angle θ of the magnetization for different angle φ, and the magnetization intensity is *M* = 0.01*t*. The corresponding out-of-plane STT (black line) is very small. The other parameters are: *λ*_*SO*_ = 0.05*t*, *N*_*x*_ = 160, *N*_*y*_ = 200 and *E*_*F*_ = 0.11*t*.

The angular dependence of the STT is shown in Fig. 6. It is found that the in-plane STT exhibits ∝ − sin(2*θ*) angular dependence and the out-of-plane STT is very small in Fig. 6(a). When we increase the magnetization intensity, the angular dependence of the in-plane STT deviates from this dependence. As can be seen, for =0.03*t*, the in-plane STT does not reach its peak at *θ* = π/4 anymore, and there is a small deviation. We found that the amplitude of the in-plane STT increases with an increase in the magnetization intensity. For example, the amplitude for *M* = 0.005*t* is around 0.7, but is increased to be around 0.9 for the amplitude *M* = 0.003*t*. Figure 6(b) shows the θ depence of the STT for several values of *φ*(0, *π*/6, *π*/3, *π*/2). It is clear that the out-of-plane STT is small for these values of φ and the magnitude of the in-plane STT decrease with the increase in φ and then increase again but with a reverse in direction. So the in-plane STT has oppositie directions for *φ* = 0 and *φ* = *π*/2, while the magnitude are very similar.

**Figure 7:**
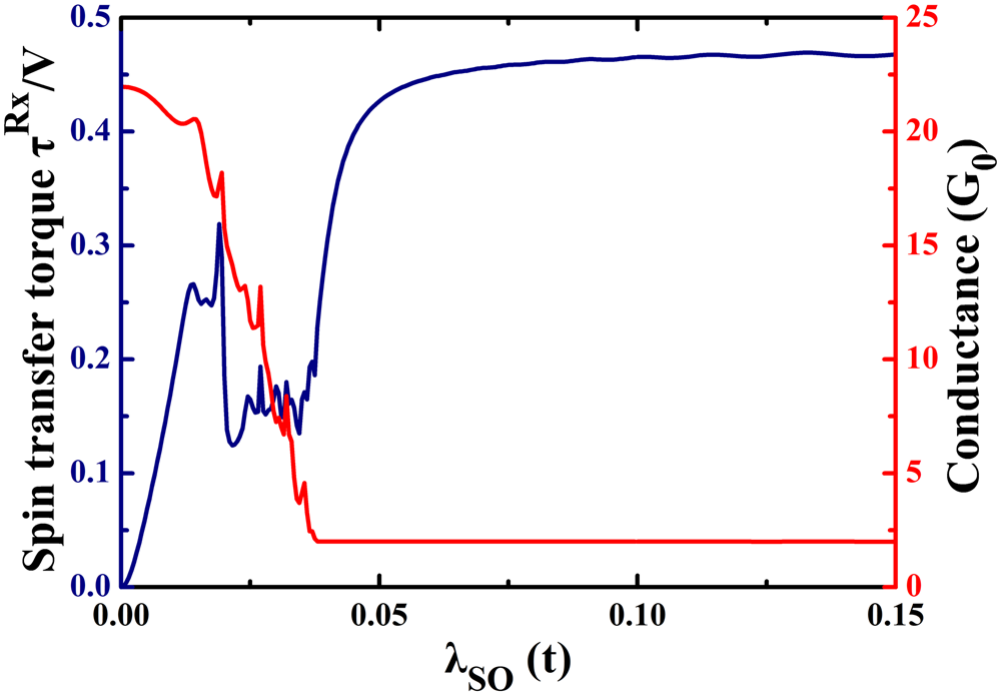
STT per unit of the bias voltage *V* in unit of *e*/4*π* and the total conductance plotted as a function of intrinsic spin-orbit interaction. The other parameters are: *M* = 0.01*t*, *N*_*x*_ = 160, *N*_*y*_ = 200, *E*_*F*_ = 0.2*t*, *θ* = 2*π*/3 and *φ* = 0.

It is known that the spin-orbit interaction can generate an energy gap in graphene, changing it into a quantum spin Hall insulator. The bulk bandgap is dependent on the value of the spin-orbit interaction, which is described by the formula Δ = 6√3*λ*_*SO*_. In Fig. 7, we set the value of Fermi energy at 0.2t and plot the STT as a function of the spin-orbit interaction. The STT and conductance oscillate with the spin-orbit interaction when *λ*_*SO*_ < 0.05*t*, because with this SOI strength the Fermi energy is not in the bandgap. When electrons move from the left lead to the right lead, there are electron scatterings, which cause the oscillations in conductance and STT. It is noted that both the STT and the conductance have very negligible changes when the Fermi energy is in the bandgap. The electrons can only move at the edges of the ribbon, and the spin-polarized dispationless currents induce a stable STT.

## Conclusions

In summary, we have investigated the current induced STT in graphene based N/TI/F heterostructures using the nonequilibrium Green’s function method. It is found that the charge current becomes spin polarized, when it flows from the left lead (N) across the quantum spin Hall insulator layer (TI), and induced STT on the magnetization of the ferromagnetic layer (F). The STT per unit of the bias voltage can reach 0.8*e*/4*π* which is comparable in magnitude to the conventional F/N/F junctions. The features of the STT can be explained through the analysis of the band structure of the right ferromagnetic graphene electrode. It is found that the STT obtained in our proposed nanostructure is immune to the changes in the geometry of the quantum spin Hall insulator, and it can not be changed by considering an asymmetric square notch or random nanopores in the quantum spin hall insulator. This offers us an efficient method to change the magnetization direction of magnetic materials without the help of a ferromagnetic layer.
